# Daily rhythms in fear extinction memory reflect structural and functional changes in a prefrontal-amygdala circuit

**DOI:** 10.1007/s00429-026-03176-8

**Published:** 2026-09-19

**Authors:** Noah R. R. Andrys, Elise A. Johnson, Ethan B. Rowe, Luke A. Millisor, Breyton K. McDole, Michael V. Baratta, Jason J. Radley, Robert L. Spencer

**Affiliations:** 1 Department of Psychological and Brain Sciences, University of Iowa, Iowa City, IA 52242 USA; 2 Iowa Neuroscience Institute, University of Iowa, Iowa City, IA 52242 USA; 3 Department of Psychology and Neuroscience, University of Colorado, Boulder, CO 80309 USA

**Keywords:** Conditioned fear, Circadian, Prefrontal cortex, Dendritic spines, FOS, Amygdala, Rat

## Abstract

Circadian rhythms exert widespread influences on learning and memory processes across diverse brain regions. The extinction of emotional memories is enhanced when training and testing occur during the circadian active phase in both rodents and humans, yet the underlying neural mechanisms remain poorly understood. Here, we examined time-of-day differences in conditioned fear extinction memory and associated neural activity within the ventromedial prefrontal cortex (vmPFC), including within a vmPFC-to-basolateral/basomedial amygdala (BLA/BMA) pathway in rats. Using an intersectional viral strategy to label vmPFC to BLA/BMA projection neurons, we found that female rats trained and tested during their active phase exhibited superior extinction recall compared to rats trained and tested during their inactive phase. The superior extinction recall was associated with greater FOS expression throughout the vmPFC, including within vmPFC to BLA/BMA projection neurons. In a follow-up experiment, we examined dendritic spine morphology in untrained female and male rats. We observed greater dendritic spine density in vmPFC to BLA/BMA neurons during the active phase compared to inactive phase, with this difference driven primarily by increased thin and stubby spine subtypes. These findings demonstrate that superior active phase extinction memory is associated with enhanced vmPFC circuit activity and corresponds to time-of-day differences in dendritic spine density within extinction-relevant neural pathways. Our results reveal a potential structural plasticity mechanism by which circadian rhythms modulate fear extinction learning and memory.

## Introduction

Circadian rhythms exert profound influence over neural function, modulating synaptic plasticity, neurotransmitter systems, and learning and memory processes across diverse brain regions (Karatsoreos et al. 2011; Smarr et al. 2014; Hartsock and Spencer 2020). Conditioned fear extinction memory exemplifies this circadian influence, with better recall in both rodents (Chaudhury and Colwell 2002; Woodruff et al. 2015, 2018; Hartsock et al. 2023, 2024) and humans (Pace-Schott et al. 2013; Zuj et al. 2016; Meuret et al. 2016) when training and testing occur during their respective daily active phases. Notably, this superior active-phase extinction recall persists in rats maintained in constant darkness (Chaudhury and Colwell 2002), indicating true circadian rather than simply light-dependent regulation.

Although the suprachiasmatic nucleus of the hypothalamus serves as the central master clock that coordinates circadian rhythms throughout the body, circadian clock genes also operate locally within individual brain regions, where they are well-positioned to regulate region-specific neuronal and functional plasticity (Guilding and Piggins 2007; Chun et al. 2015; Bering et al. 2018; Spencer et al. 2018). The ventromedial prefrontal cortex (vmPFC) is a critical node in the extinction circuit, exerting top-down inhibitory control over fear responses via projections to the amygdala (Milad and Quirk 2012; Tovote et al. 2015; Izquierdo et al. 2016; Bouton et al. 2020). We have found that superior nighttime (active phase) conditioned fear extinction memory is absent following virally mediated knockdown of clock gene expression in the rat vmPFC (Woodruff et al. 2018), indicating a regional necessity for optimal time-of-day dependent extinction. These findings suggest a role of circadian regulation of intrinsic neuroplasticity factors within the vmPFC that contribute to time-of-day differences in extinction learning.

The vmPFC and its projections to the BLA/BMA represent key neuronal components in a circuit for extinction learning and recall (Milad and Quirk 2012; Do-Monte et al. 2015; Bukalo et al. 2015; Adhikari et al. 2015; Kim et al. 2016; Bloodgood et al. 2018). Neuroplasticity within the vmPFC to amygdala pathway, including dendritic spine remodeling, is necessary for optimal extinction learning and memory (Santini et al. 2004; Burgos-Robles et al. 2007; Moench et al. 2016; Marek et al. 2019). Dendritic spines are the primary post-synaptic sites of excitatory input in cortical neurons (Nimchinsky et al. 2002), whereby changes in spine geometry and number reflect alterations in synaptic connectivity and strength underlying learning (Holtmaat and Svoboda 2009). In addition to learning-related plasticity, circadian rhythms have been shown to regulate dendritic spine dynamics in several cortical regions (Jasinska et al. 2015, 2019; Miller et al. 2022). Thus, daily circadian changes in spine morphology within the vmPFC to amygdala pathway may contribute to time-of-day differences in extinction learning.

Using an intersectional viral strategy, we examined time-of-day differences in fear extinction learning and associated neural activity in BLA/BMA-projecting vmPFC neurons (vmPFC→BLA/BMA neurons) in female rats. In a follow-up experiment in both sexes, we explored whether time-of-day differences in extinction memory are associated with time-of-day differences in dendritic spine density and morphology within vmPFC→BLA/BMA neurons of untrained rats. We hypothesized that: (1) female rats would show superior extinction memory when trained and tested at nighttime compared to daytime, similar to previous findings in males (Woodruff et al. 2015, 2018; Hartsock et al. 2023, 2024), (2) superior nighttime extinction recall would be associated with greater neural activity (FOS expression) in vmPFC→BLA/BMA neurons, and (3) in untrained rats, these neurons would show increased dendritic spine density or morphologic differences at nighttime compared to daytime. Each of these hypotheses was supported by our experimental results.

## Methods

### Animals

Sprague Dawley rats (Inotiv, West Lafayette, IN) were young adults at time of use (2–3 months old; females 238–262 g, males 328–381 g) and were housed within an AAALAC accredited animal facility at the University of Colorado Boulder. Rats were housed separately by sex in light proof and sound attenuated vivarium rooms with overhead light fixture (array of LEDs that emit ~ 3100°K white light; New Star Lighting, Chicago, IL) with light intensity of ~ 50 µW/cm^2^/~130 lx within cage as measured with a radiometer (Model ILT2400 radiometer with Model SED033/F/W detector, International Light Technologies, Peabody, MA). Rats were maintained on a 12:12 h light: dark cycle. Experimental times of day are expressed as zeitgeber time (ZT) corresponding to the number of hours after light period onset (light phase = ZT0-ZT12 and dark phase = ZT12-ZT24). Rats were acclimated to the vivarium rooms and their respective light: dark cycle for at least 2 weeks prior to experimentation. Rats (2 per cage) were housed in translucent polysulfone rat cages with a stainless steel wirebar grid top, polysulfone microisolator lid (Allentown Caging Equipment Company, Allentown, NJ) and wood chip bedding material (Teklad Sani-chip; Inotiv). Animals were given ad libitum food pellets (Inotiv Teklad 2918 rodent chow; Inotiv) and reverse osmosis chlorinated water in drinking pouches that were placed on the home cage steel wirebar grid top. Animal use complied with guidelines of the Office of Laboratory Animal Welfare and in accordance with a project specific protocol approved by the Institutional Animal Care and Use Committee at the University of Colorado Boulder.

### Viral surgeries

**Fig. 1:**
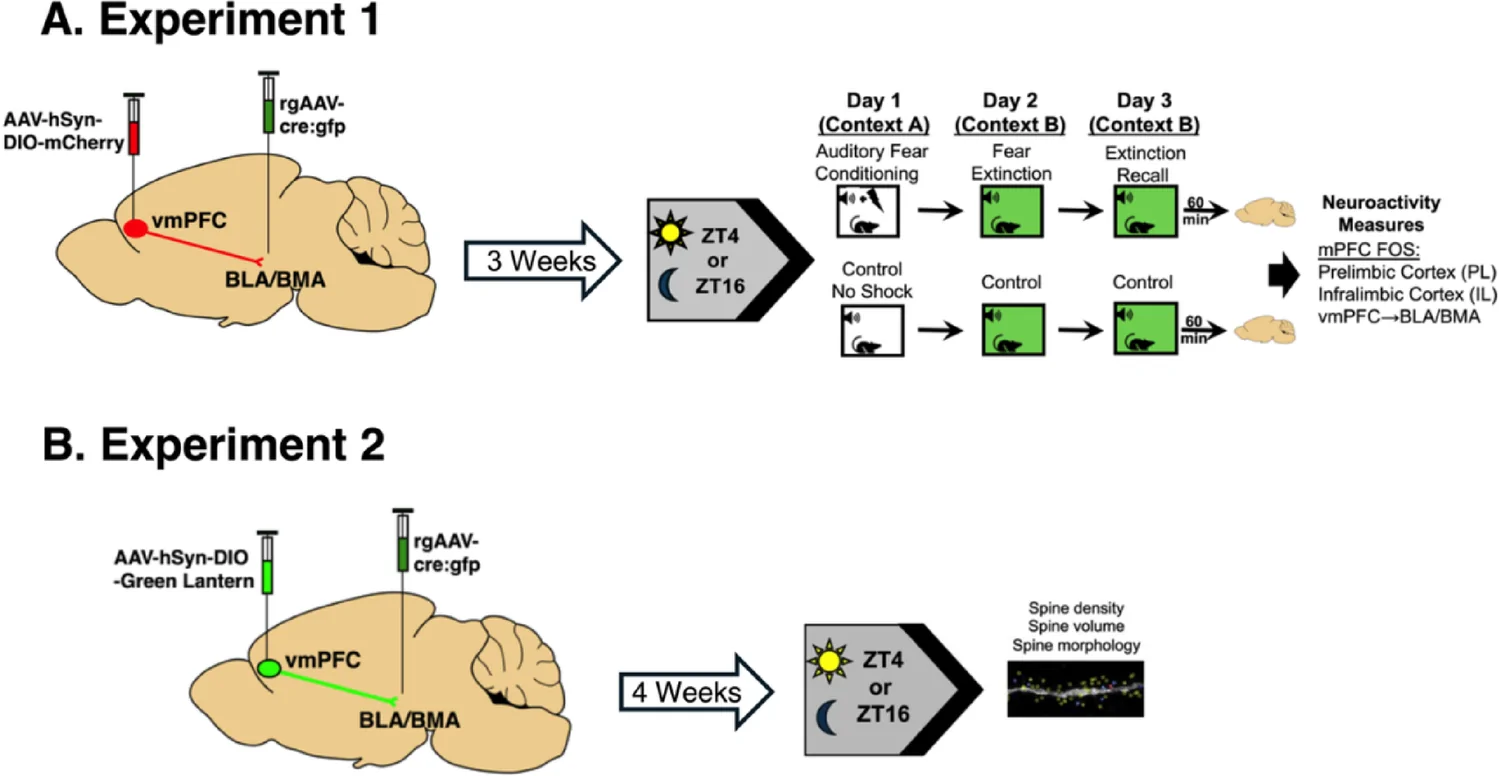
Experimental timelines. **a** Experiment 1: time-of-day comparison of conditioned fear extinction memory and neuronal activation within the ventral medial prefrontal cortex (vmPFC), including within vmPFC neurons that project to the basolateral amygdala and basomedial amygdala (BLA/BMA). Three weeks after female rats received bilateral AAV viral infusions to fluorescently label (mCherry) vmPFC neurons that project to the BLA/BMA, fear conditioned rats were trained and tested on 3 consecutive days. Control rats underwent the same 3-day test experience except that they did not receive footshock on Day 1. Rats were trained and tested at either zeitgeber time 4 (ZT4) or zeitgeber time 16 (ZT16). Rats were euthanized 60 min after the end of Day 3 testing, and brain tissue collected for postmortem measures of neuroactivity at the time of perfusion (FOS expression); *n* = 6 per comparison group (training condition X time of day). **b** Experiment 2: time-of-day comparison of basal condition dendritic spine density and morphology within vmPFC neurons that project to the BLA/BMA. Four weeks after male and female rats received bilateral AAV viral infusions to fluorescently label (mGreenLantern) vmPFC neurons that project to the BLA/BMA, untrained rats were perfused under basal conditions at either ZT4 or ZT16. Brain tissue was collected and processed for labelled vmPFC neuron dendritic spine measures; *n* = 8 per time of day (4 female and 4 male rats)

We used an intersectional viral strategy to fluorescently tag vmPFC neurons that project to the BLA/BMA. For this strategy, a retrograde AAV virus carrying the Cre-recombinase (Cre) transgene was bilaterally injected into the BLA/BMA. In addition, an AAV virus carrying Cre-dependent mCherry or Cre-dependent mGreenLantern transgenes was bilaterally injected into the vmPFC. Thus, the fluorophores mCherry or mGreenLantern were only expressed in vmPFC neurons that had taken up the retrograde virus from the BLA/BMA and subsequently express Cre (Fig. 1). Rats were deeply anesthetized with isoflurane inhalant and given buprenorphine (0.2 mg/kg s.c) as a general anesthetic. Holes (~ 1 mm diameter) were drilled through the skull over the microinfusion sites with a hand-held motor driven Dremel drill. The virus was delivered via a Hamilton syringe (31 ga beveled needle) attached to a stereotaxic pump (0.1 µl/min; UMP3-1; World Precision Instruments). The syringe needle remained in place for an additional 10 min after infusion. After surgery rats were given ibuprofen in their drinking water (0.4 mg/ml) for three days to reduce postoperative pain and inflammation. For both Experiment 1 and Experiment 2, rats were given bilateral injections (0.5 µl) of the Cre-expressing retrograde virus (AAVrg-hSyn-eGFP-CRE; Addgene) in the basal amygdala targeting the BLA/BMA (coordinates— AP:−2.3 mm; ML:±4.0 mm; DV: −9.1 mm; (Paxinos and Watson 2007). Rats were also given bilateral injections (0.75 µl) of Cre-dependent expression virus (Experiment 1: AAV-hSyn-DIO-mCherry; Addgene; Experiment 2: AAV2/1.EF1a.DIO.mGreen Lantern; 1:3 dilution; Neurophotonics) in the vmPFC targeting the infralimbic cortex (IL; coordinates— AP:+2.7 mm; ML:±0.5; DV:−5.0 mm; (Paxinos and Watson 2007). In pilot experiments for Experiment 2, we found that the mGreenLantern expressing virus when diluted 1:3 in sterile saline provided strong immunofluorescence in PFC pyramidal neuron dendrites while allowing for reasonable visible separation of individual neuronal segments from those of other nearby neurons.

## Experiment 1: time-of-day comparison of conditioned fear extinction memory and neuronal activation within the vmPFC

Twenty-four female rats were randomly divided into four treatment groups (*n* = 6), designated as (1) conditioned fear-ZT4, (2) conditioned fear-ZT16, (3) control-ZT4, and (4) control-ZT16 (Fig. 1a). Three to four weeks prior to behavioral testing rats received stereotaxic surgical infusion of AAV viruses to fluorescently label (mCherry) vmPFC→BLA/BMA neurons. Rats then underwent 3 days of behavioral testing, and 60 min after the third test day session brains were collected for subsequent FOS immunohistochemistry.

### Behavioral testing

The conditioned fear groups were given 3 consecutive days of a conditioned fear/extinction training paradigm (Woodruff et al. 2015, 2018; Hartsock et al. 2024). On Day 1 (conditioned fear training) rats were placed in Context A for the first time and given 5 min of baseline acclimation to the test chamber, followed by 3 tone-shock pairing trials using a delayed auditory conditioned fear procedure (Woodruff et al. 2018). Auditory tone (80 dB, 3 kHz) durations were 30 s and co-terminated with 2 s of foot shock (0.85 mA). Intertrial intervals (ITI) were 120 s. On Day 2 (conditioned fear recall and extinction training), rats were placed in Context B for the first time and given 3 min of baseline acclimation, followed by 15 trials of auditory tone-alone (same parameters as Day 1). Intertrial intervals on Day 2 were 90–120 s (pseudo-randomly determined for each trial). On Day 3 (extinction recall) rats were returned to Context B and given 4 additional tone alone trials as described for Day 2. The control groups were given 3 consecutive days of testing that were identical to that of the conditioned fear groups with the exception that on Day 1 the control groups did not receive foot shock (i.e., no fear conditioning). Fear conditioned groups and control groups were trained and tested either 4 h into their light phase (ZT4) or 4 h into their dark phase (ZT16). For rats that were tested during their light/inactive phase, lights were on at 0600 h, and for rats tested during their dark/active phase, lights were on at 2300 h. These light-dark schedules permitted behavioral testing of rats centered around 1000 h (ZT4 groups) and 1500 h (ZT16 groups).

### Behavioral test chambers

Behavioral testing occurred within fear conditioning chambers housed inside light-tight boxes (MED-VFC-OPTO-USB-R, Med Associates). The floor of the chambers had a grid of stainless-steel bars through which electric current could be delivered. The chambers also include an auditory speaker for delivering auditory tone. Internal lighting conditions in the chamber consisted of either white light exposure (ZT4 training and testing conditions) or near-infrared light (LED peak 935 nm) exposure (ZT16 training and testing conditions). For Context A conditions, the standard chamber as described above remained unmodified. For Context B conditions, a solid plastic floor insert was placed over the metal grids, and a curved plastic wall was inserted which changed the internal shape of the chamber from a rectangular shape to a semicircle shape. Light conditions, auditory tone presentations and foot shock delivery in the chambers were controlled by automated software, and each session was video recorded (Video Fear Conditioning software, Med Assoc). The amount of freezing (fear conditioned groups) or total activity (control groups) was quantified by automated software (VideoFreeze software, Med Assoc).

Rats were transferred from their home cage room to the test room in light-tight opaque plastic tubs with lids. For rats that were tested at ZT16, lights remained off in their home cage room and were turned off in the test room during transfer of rats into and out of the transfer boxes and the behavioral test boxes. This transfer procedure ensured that the rats received no incidental white light exposure during the test procedure. We have previously reported that this transfer procedure as well as the near infrared light exposure during testing does not activate the suprachiasmatic nucleus master clock (Stritzel et al. 2025).

### Tissue processing for FOS image analysis

Sixty minutes after the onset of Day 3 testing, rats were deeply anesthetized (pentobarbital, 200 mg/kg) and perfused with 400 ml of 0.01 M phosphate buffered saline (pH 7.4) followed by 400 ml of 4% paraformaldehyde in 0.1 M phosphate buffer (PB). Brains were then post-fixed for 24 h in 4% paraformaldehyde in 0.1 M PB and then transferred to a solution of 30% sucrose in PB for 72 h. Brains were then snap frozen in isopentane (cooled with dry ice to ~ −30 ° C) and stored in a −70 ° C freezer.

Frozen brains were sliced (35 μm thick coronal sections) on a cryostat (Leica model 1850), and 4 series of 1 in 6 sections were collected at the level of the vmPFC (between approximately + 4 and + 2 mm anterior to bregma) and the BLA/BMA (between approximately − 2 and − 3.5 mm posterior to bregma) according to anatomical landmarks (Paxinos and Watson 2007), and stored at −20 °C in tissue wells containing cryoprotectant. Tissue sections subsequently underwent immunofluorescence staining for FOS protein. Tissue sections were incubated overnight in primary antibody (rabbit anti-rFOS[4–17] serum, code PBL #6972; 1:1500 dilution of stock; gift of Paul E Sawchenko), and the next day incubated for 1 h in secondary antibody (Cy5 conjugated goat anti rabbit; 1:1000 dilution of stock; cat# A10523, Invitrogen, Thermo Fisher Scientific). Tissue sections were mounted onto ColorFrost Plus microscope slides (Fisherbrand, Fisher Scientific) and cover-slipped with mounting media (ProLong Gold antifade mountant, Invitrogen).

Tiled three-channel images of regions of interest were collected with 10X objective using green, red and far-red fluorescent filter sets to image GFP, mCherry, and FOS, respectively (Zeiss Axio Imager M1 epifluorescent microscope, Axiocam 305 monochrome camera, and ZEN software; Carl Zeiss Microscopy). Exposure times for each channel were held constant for all image acquisition. Cell counts of total FOS in the prelimbic (PL) and IL cortex, and FOS colocalization with mCherry in the IL cortex were performed by an individual (blind to treatment conditions) using the QuPath (version 0.6.0-rc3) open-source software for bioimage analysis (Bankhead et al. 2017). A minimum of 3 sections per brain were analyzed and the cell count density (# of fluorescent positive cells per mm^2^) was averaged across brain sections for each rat.

## Experiment 2: time-of-day comparison of dendritic spine density and morphology within the vmPFC to BLA/BMA pathway

Experiment 2 examined whether there are time-of-day differences in dendritic spine density in vmPFC→BLA/BMA neurons of untrained rats. We included both female and male rats, balanced across time of day, with 8 female and 8 male rats randomly assigned to tissue collection under basal conditions at ZT4 or ZT16 (*n* = 8/time-point) (Fig. 1b). Due to the logistical limitations of performing these spine measures in large samples, the small number of females and males used for each time-point (*n* = 4/sex) was not sufficiently powered to treat sex as an independent variable at the individual animal level for analysis of spine density. However, inclusion of both sexes permitted exploratory consideration of sex as a biological variable in measures of spine volume, for which the unit of measure was individual spines. Three to four weeks prior to tissue collection, rats received stereotaxic surgical infusion of AAV viruses to fluorescently label (mGreenLantern) vmPFC→BLA/BMA neurons (see Methods: Viral Surgeries). The mGreenLantern expressing virus has been found to be effective for in vivo neuronal labelling of postmortem dendritic and spine morphological analyses (Campbell et al. 2020).

Six days prior to perfusion, rats underwent a sham adrenalectomy surgery (Woodruff et al. 2015) so that these data would be more suitable for direct comparison to prior and subsequent related behavioral and morphological experiments that include adrenalectomy and sham adrenalectomy treatment groups (Woodruff et al. 2015, 2018; Anderson et al. 2016). Because all rats in Experiment 2 received the identical sham adrenalectomy, any effect of the procedure was balanced across the ZT4 and ZT16 groups and cannot account for the observed time-of-day difference in spine density. Sham adrenalectomy surgery was performed under isoflurane anesthesia. Bilateral incisions were made through the dorsolateral skin and peritoneal wall near the kidney as in the adrenalectomy procedure of Woodruff et al. (Woodruff et al. 2015), but the adrenal glands were left intact. The brief (~ 20 min) surgical procedure was performed during the rat’s inactive/light phase. Rats were then left undisturbed until 6 days later at the designated time of perfusion (ZT4 or ZT16) following the same transcardial perfusion procedure described above for Experiment 1. Brains were extracted and placed in vials containing 4% paraformaldehyde in 0.1 M PB and then shipped overnight unfrozen to the University of Iowa for subsequent processing for dendritic spine measurements.

### Tissue processing, imaging and dendritic spine morphometric analyses

Brains were sectioned coronally in 250 μm thick slabs through the prefrontal cortex, and 75 μm thick sections were collected through the remainder of the forebrain, using an oscillating tissue slicer (VT-1000 S, Leica). Sections were collected and stored in 0.1 M PB containing 0.1% sodium azide at 4 °C. An experimenter unaware of the treatment condition for each animal performed neuronal reconstructions and data analyses. Virally infected pyramidal neurons in vmPFC bearing axonal projections to the BLA/BMA were identified by the expression of the fluorophore, mGreenLantern. Accurate placement of viral injections in the basal amygdala was confirmed prior to inclusion of cases into the dendritic spine analysis. mGreenLantern expressing dendritic segments in vmPFC were randomly selected for imaging from both apical and basal arbors, with limiting criteria that they: (1) did not overlap with other dendritic segments, (2) displayed complete filling of mGreenLantern (Campbell et al. 2020), and (3) were > 100 μm from neuronal somata. Portions of segments within 5 μm of a branch point or from the terminal end of the dendrite were excluded from analysis.

*z*-Stacks were collected on a Leica SP8 confocal laser-scanning microscope equipped with an argon laser and a 100X, 1.4 NA oil-immersion objective, using voxel dimensions of 0.05 × 0.05 × 0.05 µm^3^. Settings for pinhole size (1 airy disc), gain, and offset were optimized initially and then held relatively constant throughout the study to ensure that all images were digitized under similar illumination conditions. Dendrites were digitally rotated and imaged at a resolution of 256 × 1024 pixels and then deconvolved using Huygens Essential software 22.10 (Scientific Volume Imaging B.V., Hilversum, Netherlands) with automated express deconvolution settings. Dendritic segments were traced and spine morphology was analyzed using Neurolucida 360 (MBF Bioscience, Williston, VT, USA) via user-guided semi-automated spine tracing.

Spine volume, head diameter, neck diameter, and spine length were automatically generated, and these values were used to classify spines into subtypes. Spines were designated as thin or mushroom if the ratio of the head diameter-to-neck diameter was > 1.1. If their ratio exceeded this value, spines with a maximum head diameter > 0.35 μm were classified as mushroom, or else were classified as thin. Spines with head-to-neck diameter ratios < 1.1 were also classified as thin if the ratio of spine length-to-neck diameter was > 2.5; otherwise, they were classified as stubby. A fourth category, filopodial spines, exhibited a long (> 3 μm) and thin shape with no enlargement at the distal tip. Since these were very seldom observed, they were classified as thin subtypes. Data from the dendritic spine analysis were manually evaluated by the experimenter for each optical stack to verify accurate subtype classifications for dendritic spines.

Spine density was calculated as the number of spines per micron of dendrite length. All spines detected and classified by Neurolucida 360 were included in density measurements, with no exclusions based on spine volume. For population analyses of spine volume distributions, structures with volumes < 0.01 μm³ (approaching resolution limits) or > 1.5 μm³ (likely representing dendritic shaft segments or segmentation errors) were excluded, representing < 0.3% of all detected structures. Mean spine volumes and cumulative frequency distributions were calculated from the filtered data (0.01–1.5 μm³ range).

An average of 7.7 dendritic segments were imaged in the vmPFC per rat. The mean length of dendritic segments included in the analysis was 48.8 μm. In total, 9,281 spines were included in the analysis, with averages of 320 spines per sex per time-point, and 580 spines per rat. Density values were obtained as the number of spines per unit µm of dendritic segment from each animal.

### Statistics

Statistical analyses were performed using the software packages IBM SPSS Statistics 29.0.2.0 (IBM, Armonk, NY), GraphPad Prism 10.4.0 (Graphpad Software, San Diego, CA) and R software (R Core Team, 2025). Multifactorial analysis of variance (ANOVA) was used to determine statistical significance for experimental factors in each experiment with more than two comparison groups. For experiment 1, behavioral data for each of the 3 test days were analyzed separately for fear conditioned rats (% time freezing) and control rats (activity counts) using mixed model ANOVA. Time of Day (ZT) was a between-subjects factor. Trial (Day 1 and Day 3) or Trial Block (average of 3 trials; Day 2) was a within-subjects factor, and Trial Epochs (Tone and Intertrial Interval) were additional within-subjects factors nested within Trial for Day 1 and Day 3. Post hoc tests of ZT differences at individual time-points were performed using Bonferroni correction of multiple comparisons across a family of time-points (e.g. trial blocks, tones or intertrial intervals). ZT differences at pre-tone baselines were assessed by Student’s independent t-test. Time-of-Day differences in FOS expression were analyzed by 2-way ANOVA with Time-of-Day (ZT) and Training Condition as between-group factors. Follow-up exploratory analyses of ZT differences within Training Condition (Fear Conditioned or Control) were performed using Bonferroni correction of multiple comparisons or the relatively conservative Student’s independent t-test with Welch correction, as indicated in the Results section. For experiment 2, spine density measures were analyzed using Student’s independent t-test (with Welch correction in cases of unequal variance; *n* = 8/time point). In keeping with NIH guidelines concerning sex as a biological variable (NIH 2025), spine density values for each sex are reported in the relevant graphs. Spine-volume distribution analyses included both sex and time-of-day as between group factors, as the unit of analysis (the individual spine; thousands per group) provides the resolution to detect population-level differences; these sex-stratified volume analyses are considered exploratory. Population analyses of spine volume were analyzed via comparison of cumulative frequency distributions using the Kolmogorov–Smirnov (K-S) test with GraphPad Prism. Since the K-S test allows comparisons of only two distributions, we performed a correction for multiple comparisons using an expanded Bonferroni approximation (Brittain 1987). Effect sizes are reported as Cohen’s d for t-tests and D statistic for K-S tests. For K-S tests, we used an alpha level of 0.01, and with 4 comparisons, the Bonferroni-corrected threshold was *p* <.0025. For all other statistical tests the alpha level was 0.05. Data graphs depict treatment group (mean + SEM), with superimposed individual subject data points for FOS expression and spine measures.

## Results

### Experiment 1: time-of-day comparison of conditioned fear extinction memory and neuronal activation within the vmPFC

#### Rats trained and tested at ZT16 have superior extinction recall

We have previously found that male rats have superior conditioned fear extinction recall when trained and tested at ZT16 compared to ZT4 (Woodruff et al. 2015, 2018; Hartsock et al. 2024). In this experiment, we examined for the first time time-of-day differences in extinction recall in female rats. Importantly, we included a control group of rats that were exposed to the exact same 3-day training and test conditions, with the one exception that the control rats did not receive foot shock on Day 1. This allowed us to rule out the possibility that time-of-day differences in freezing behavior or vmPFC activity levels could be simply a reflection of time-of-day differences in general behavioral activity levels under these test conditions.

**Fig. 2:**
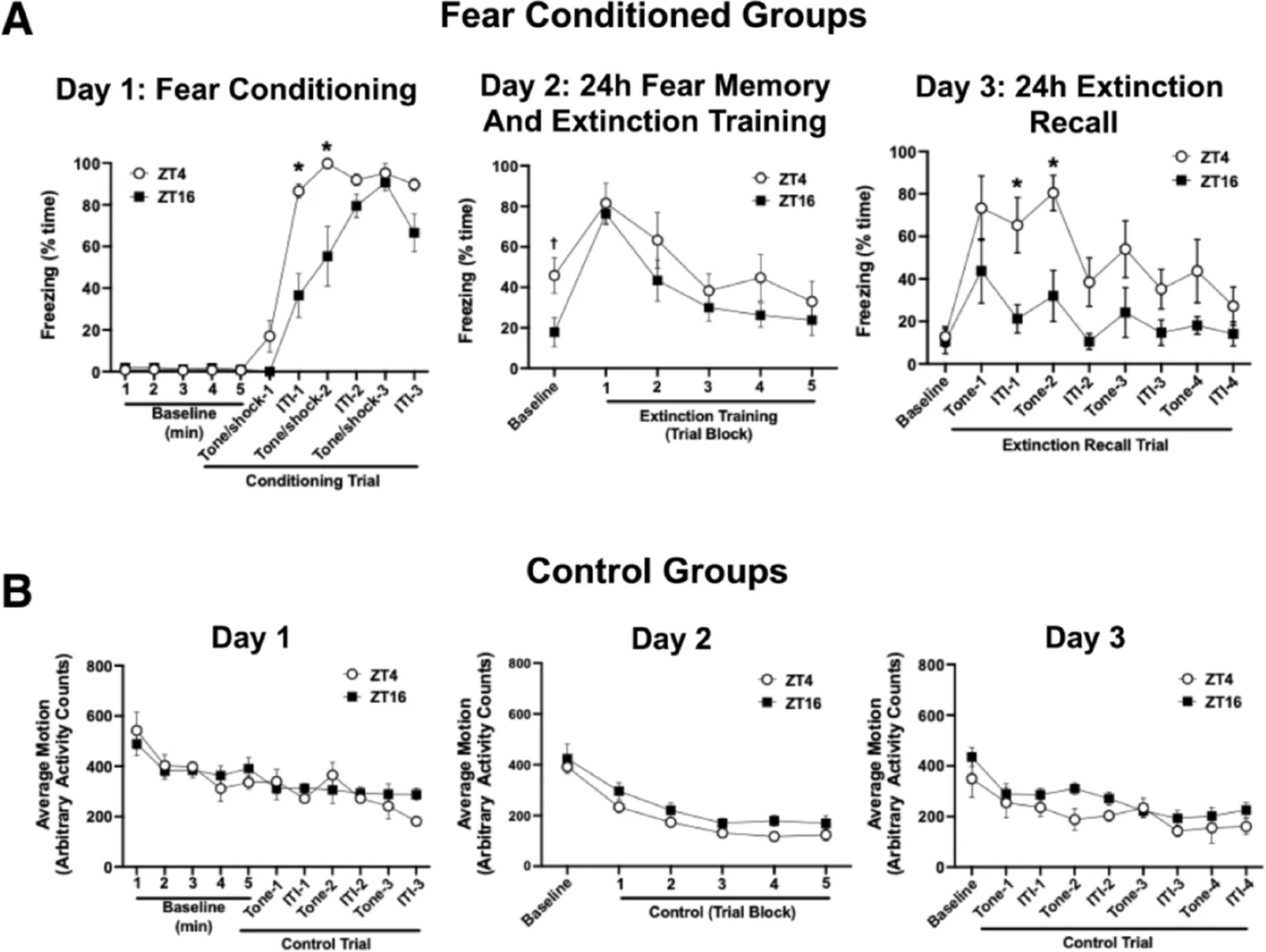
Rats trained and tested at ZT16 have superior extinction recall. **a** Fear conditioned rats (3 tone/shock pairings on Day 1 in Context A), trained and tested each day at ZT4 or ZT16 had similar 24 h cued conditioned fear recall to tone-alone on Day 2 in Context B (freezing during Trial Block 1) and similar within session extinction (Day 2 Trial Blocks 2–5). However, when rats were returned to Context B on Day 3 and again presented with tone-alone, rats trained and tested at ZT16 had superior extinction recall (decreased freezing) compared to rats trained and tested at ZT4. **b** Control rats (no shock exposure on Day 1), trained and tested each day at ZT4 or ZT16 had similar activity levels regardless of time of day for each of the 3 test days. ITI = intertrial interval; *n* = 6; †indicates time of day difference for the pre-tone baseline period, *p* <.05, Student’s t-test. *indicates time-of-day difference for that specific test trial component, Bonferroni correction for multiple comparisons, p critical < 0.017 for Day 1, p critical < 0.01 for Day 2, and p critical < 0.013 for Day 3

On Day 1 (Context A), fear conditioned rats at both times of day displayed strong freezing responses to 3 tone-footshock pairings (Fig. 2a). However, ZT16 rats displayed less total freezing time than ZT4 rats that varied across trials (ZT: F_1,10_ = 15.90, *p* =.003; Tone Trial X ZT: F_2,20_ = 4.27, *p* =.029; ITI X ZT: F_2,20_ = 7.97, *p* =.003). This time-of-day difference was most pronounced (Bonferroni multiple comparisons tests) during the first intertrial interval (t_10_ = 4.52, *p* =.001) and the second tone presentation (t_10_ = 3.10, *p* =.01). On Day 2 (Context B), rats trained and tested at both times of day showed strong and comparable levels of cued conditioned fear recall to tone-alone (no time-of-day difference in freezing during Trial Block 1, t_10_ = 0.46, *p* =.66, Bonferroni multiple comparisons test). Both groups also exhibited similar within session extinction learning across trial blocks 2–5 (overall Trial effect: F_4,40_ = 33.18, *p* <.001; overall ZT effect: F_1,10_ = 1.16, *p* =.308; Trial X ZT effect: F_4,40_ = 0.83, *p* =.51). We also note that ZT4 rats exhibited more freezing behavior on Day 2 during the pre-tone baseline period (Student’s t-test, t_10_ = 2.48, *p* =.03), perhaps reflecting greater generalization of conditioned fear of this group to context B or to other aspects of the testing experience. Notably, On Day 3 (Context B), there was a pronounced time-of-day difference in conditioned fear extinction recall, with superior recall (less freezing) in rats trained and tested at ZT16 (overall ZT effect: F_1,10_ = 6.29, *p* =.031).

Control rats (no shock exposure on Day 1) never displayed an instance of stereotypic freezing behavior across the three days of testing. Consequently, we quantified general activity levels rather than freezing behavior in control rats to test whether there were time-of-day differences in general activity levels of rats in these test situations in the absence of fear conditioning. Control rats showed no altered activity levels in response to tone stimuli and no time-of-day differences in activity levels across all three days (*p* >.05 for all relevant ANOVAs) (Fig. 2b).

#### Rats trained and tested at ZT16 have greater FOS expression in vmPFC, including within IL→BLA/BMA neurons, after extinction recall

**Fig. 3:**
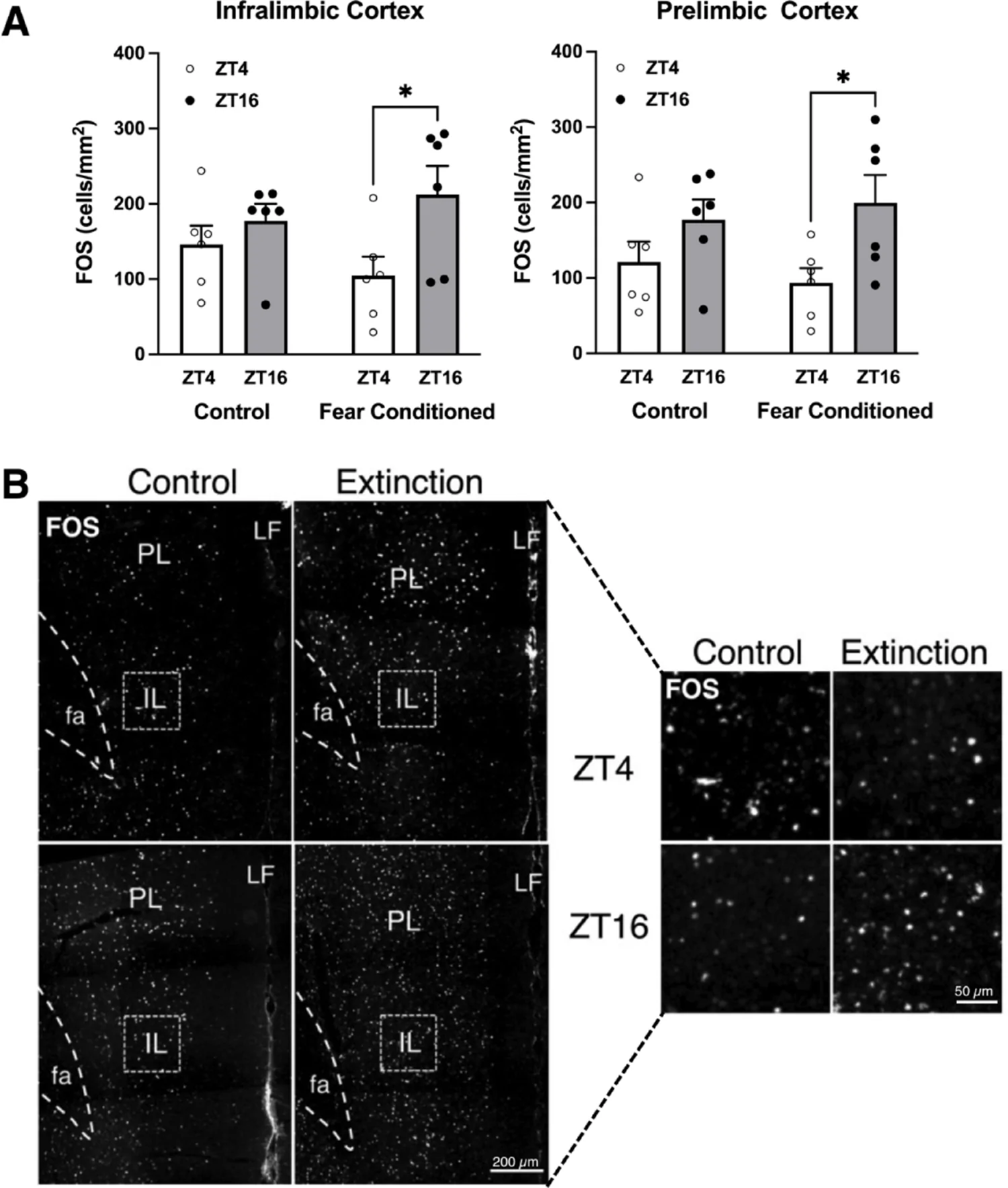
Rats trained and tested at ZT16 have greater vmPFC FOS expression after extinction recall. **a** Fear conditioned rats trained and tested at ZT16, had greater FOS expression in the infralimbic cortex (IL) and prelimbic cortex (PL) after Day 3 extinction recall testing compared to fear conditioned rats trained and tested at ZT4; *Bonferroni adjusted *p* <.05, exploratory Bonferroni multiple comparison test of ZT within Training Condition. **b** Representative photomicrographs of FOS immunofluorescence expression (white neuronal nuclei) in vmPFC of control and fear conditioned rats at ZT4 and ZT16. The right hand set of images are enlarged portions (white dashed line boxes within the IL region) of the left hand set of images. Note higher density of FOS immunoreactive cells in the ZT16 fear condition. fa, forceps anterior, corpus callosum; LF, longitudinal fissure

On Day 3, rats were perfused 60 min after the onset of testing (extinction recall test for fear conditioned rats), and brains removed for subsequent FOS expression analysis. In both the IL cortex and PL cortex there was an overall significant ZT effect (IL: F_1,20_ = 6.04, *p* =.02; PL: F_1,20_ = 8.20, *p* =.01), but no overall difference between Fear Conditioned and Control rats (IL: F_1,20_ = 0.01, *p* =.91; PL: F_1,20_ = 0.01, *p* =.93) or an interaction between ZT and Training Condition (IL: F_1,20_ = 1.85, *p* =.19; PL: F_1,20_ = 0.77, *p* =.39). In a follow-up exploratory test of whether the reliability of the ZT effect differed between Fear Conditioned and Control rats, Bonferroni Multiple Comparisons test revealed greater FOS expression at ZT16 in fear conditioned rats (IL: t_20_ = 2.70, *p* =.027; PL: t_20_ = 2.64, *p* =.031) but not in control rats (IL: t_20_ = 0.78, *p* =.89; PL: t_20_ = 1.40, *p* =.35; Fig. 3).

**Fig. 4:**
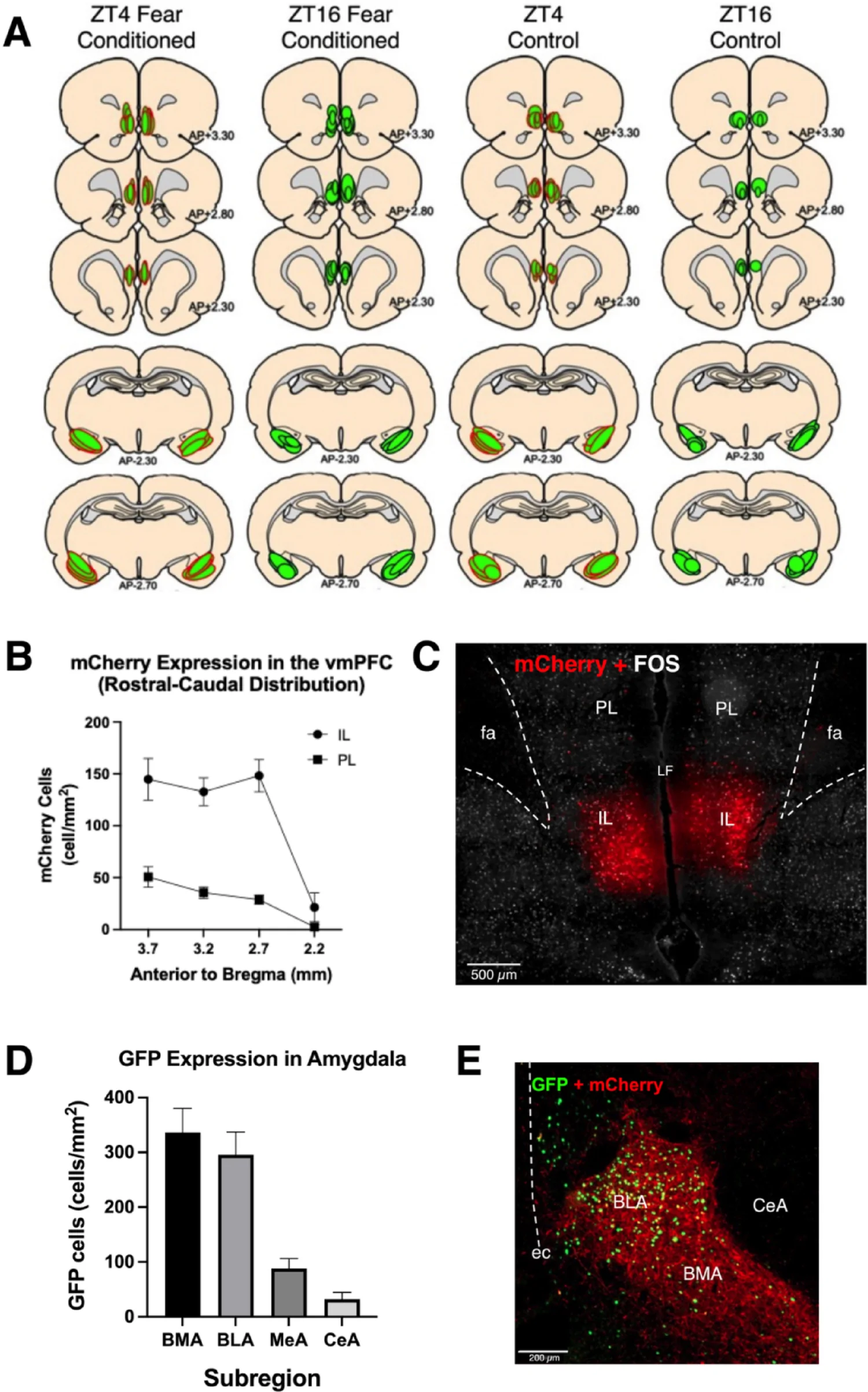
Experiment 1 validation of virally mediated fluorescent labelling of vmPFC neurons that project to the BLA/BMA. We used stereotaxic coordinates to target bilateral AAV retrograde-Cre virus (GFP reporter) infusions into the BLA/BMA, and bilateral AAV Cre-dependent mCherry virus infusions into the infralimbic cortex (see Methods). **a** Illustrations of rat brain coronal sections showing for individual rats (oval contours) in each treatment group the approximate mCherry expression in vmPFC (upper 3 sections) and approximate amygdala retrograde-Cre injection sites (lower 2 sections) based on localized expression of the AAV retrograde-Cre virus (GFP reporter). Anteroposterior (AP) coordinates for each coronal illustration are displayed relative to bregma. **b** Plotting average mCherry expression cell counts for all rats, we found that mCherry expression was strongly expressed throughout the rostral extent of the infralimbic portion (IL) of the vmPFC, with modest expression in the rostral extent of the prelimbic (PL) portion of the vmPFC. **c** Representative epifluorescent coronal plane photomicrograph (10X objective) showing example of bilateral mCherry labeled neurons centered in the IL, with FOS immunofluorescent labeling (white neuronal nuclei) throughout tissue serving to illustrate some relevant anatomical landmarks (fa, forceps anterior, corpus callosum; LF, longitudinal fissure). **d** Strong GFP labeled neurons in the amygdala were primarily found in the BLA and BMA portions of the amygdala. There was modest GFP expression in the medial amygdala (MeA) and central nucleus of the amygdala (CeA). **e** Representative epifluorescent coronal plane photomicrograph (20 X objective) showing example of GFP labeled neurons centered within the basal amygdala (BLA/BMA) surrounded by strong Cre-dependent mCherry labelling of vmPFC neuronal axonal processes. White dashed line shows approximate location of the external capsule (ec)

We next examined FOS expression specifically within IL→BLA/BMA neurons. Cre-dependent mCherry labelled neurons were preferentially expressed in the rostral infralimbic cortex (Fig. 4a-c). Retro-Cre viral injections (GFP reporter surrounding injection site) were localized primarily to BLA and BMA subregions (Fig. 4a, d), and we observed strong mCherry terminal expression in the BLA and BMA (Fig. 4e).

**Fig. 5:**
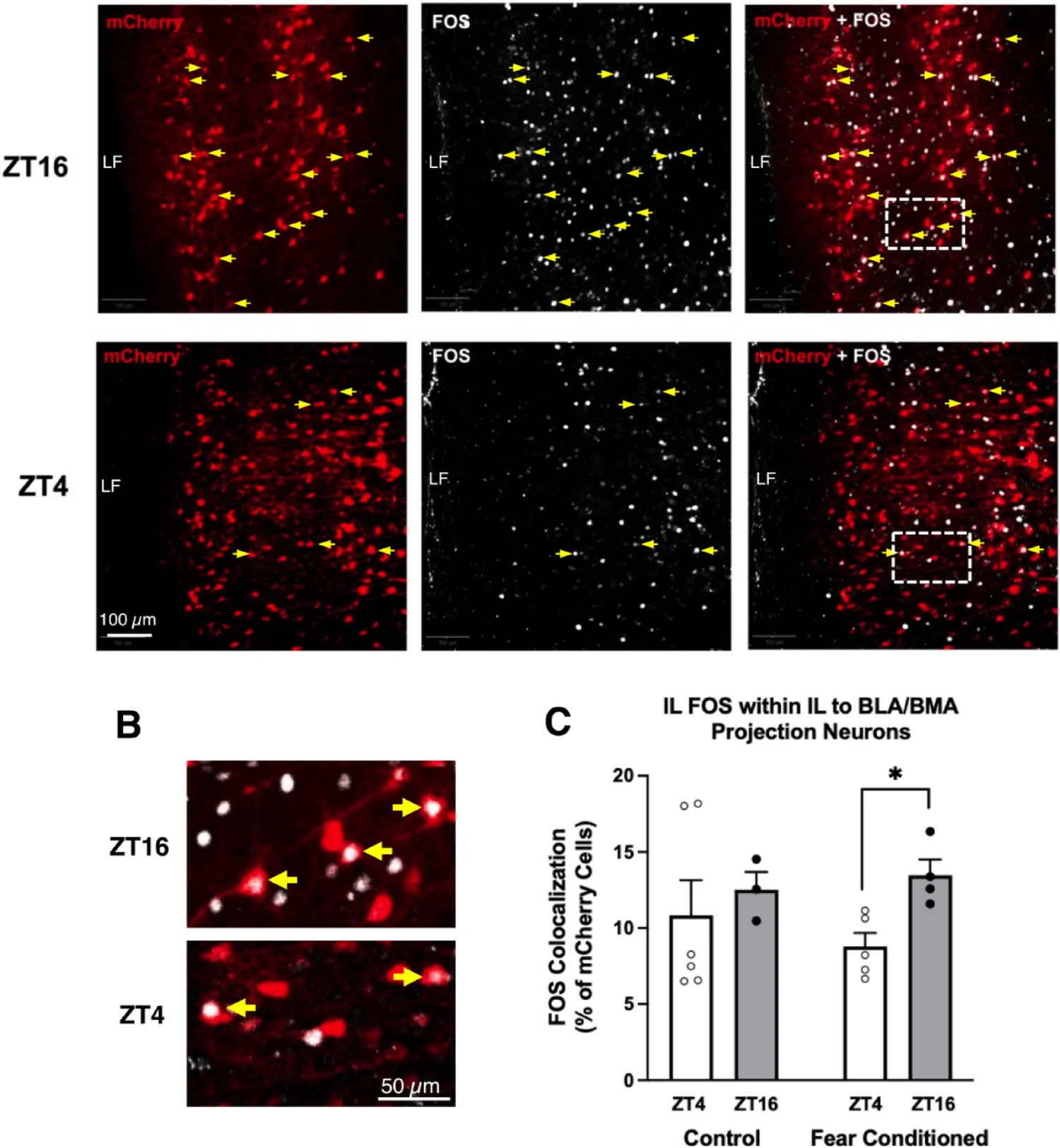
Rats trained and tested at ZT16 have greater FOS expression in IL to BLA/BMA projection neurons after extinction recall. **a** Representative epifluorescent coronal plane photomicrographs (10 X objective) of mCherry expression (red) and FOS immunoreactivity (white) in IL of fear conditioned rats at ZT4 and ZT16. Arrows point to some examples of FOS immunolabelling within nuclei of mCherry labeled IL to BLA/BMA projection neurons. LF, Longitudinal Fissure. **b** Enlarged portion of (**a**) mCherry + FOS co-expression (white dashed line boxes). Arrows point to the same examples of FOS immunolabelling within nuclei of mCherry labeled IL to BLA/BMA projection neurons that are depicted in the white boxes in (**a**). **c** Fear conditioned rats trained and tested at ZT16 had greater FOS expression in IL to BLA/BMA projection neurons after Day 3 extinction recall testing compared to fear conditioned rats trained and tested at ZT4; *p <.05, exploratory Student’s t-test with Welch correction

Although a relatively low percentage of IL→BLA/BMA neurons expressed FOS after extinction recall, there was greater FOS expression in those neurons of rats trained and tested at ZT16 (13.5%) than ZT4 (8.8%), t_6.5_ = 3.44, *p* =.01, with no time-of-day difference in control rats, t_6.8_ = 0.65, *p* =.54 (Student’s t-tests with Welch’s correction; Fig. 5).

### Experiment 2: time-of-day comparison of dendritic spine density and morphology within the vmPFC to BLA/BMA pathway of untrained rats

#### Greater spine density in vmPFC→BLA/BMA neurons at ZT16 is driven by thin and stubby spine subtypes

**Fig. 6:**
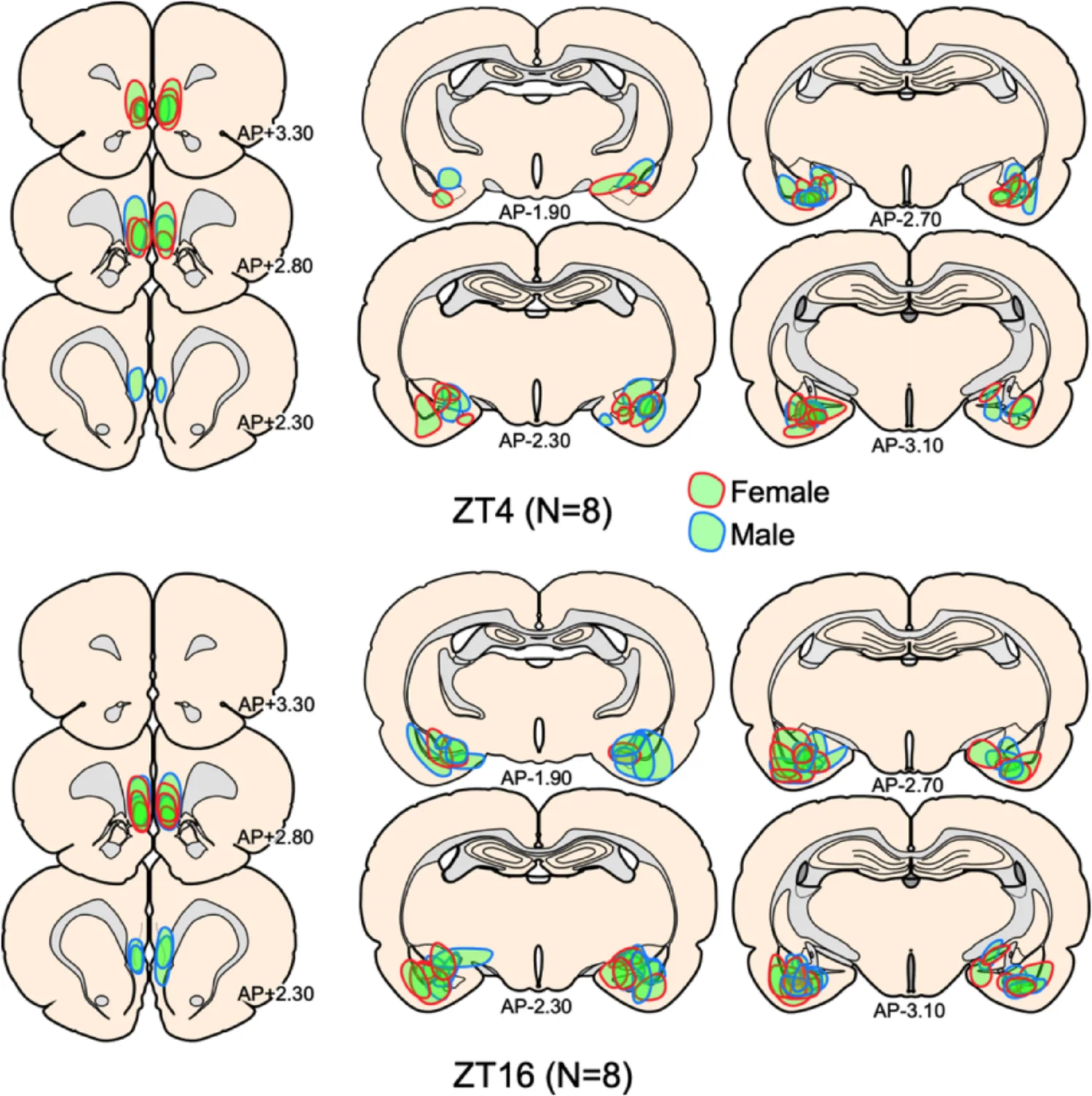
Experiment 2 validation of intersectional viral strategy for labeling vmPFC to BLA/BMA projection neurons. Injection sites are depicted using illustrations of rat brain coronal sections, showing approximate mGreenLantern expression based on injection of AAV-DIO-mGreenLantern in vmPFC (left), and AAV-retro-Cre in BLA/BMA (right). Somatic and dendritic expression in vmPFC neurons is based on the degree to which retrograde transport of AAV-retro-Cre from BLA/BMA is co-expressed in neurons bearing AAV-DIO-mGreenLantern. Anteroposterior (AP) coordinates for each coronal illustration are displayed relative to bregma. Individual rats are indicated by contours of red (female rats) and blue (male rats). Each animal is represented by multiple contours corresponding to the extent of viral expression across the rostro-caudal series of sections; contours at the rostral or caudal extremes may extend beyond the core target, but every included case showed expression overlapping the BLA/BMA

**Fig. 7:**
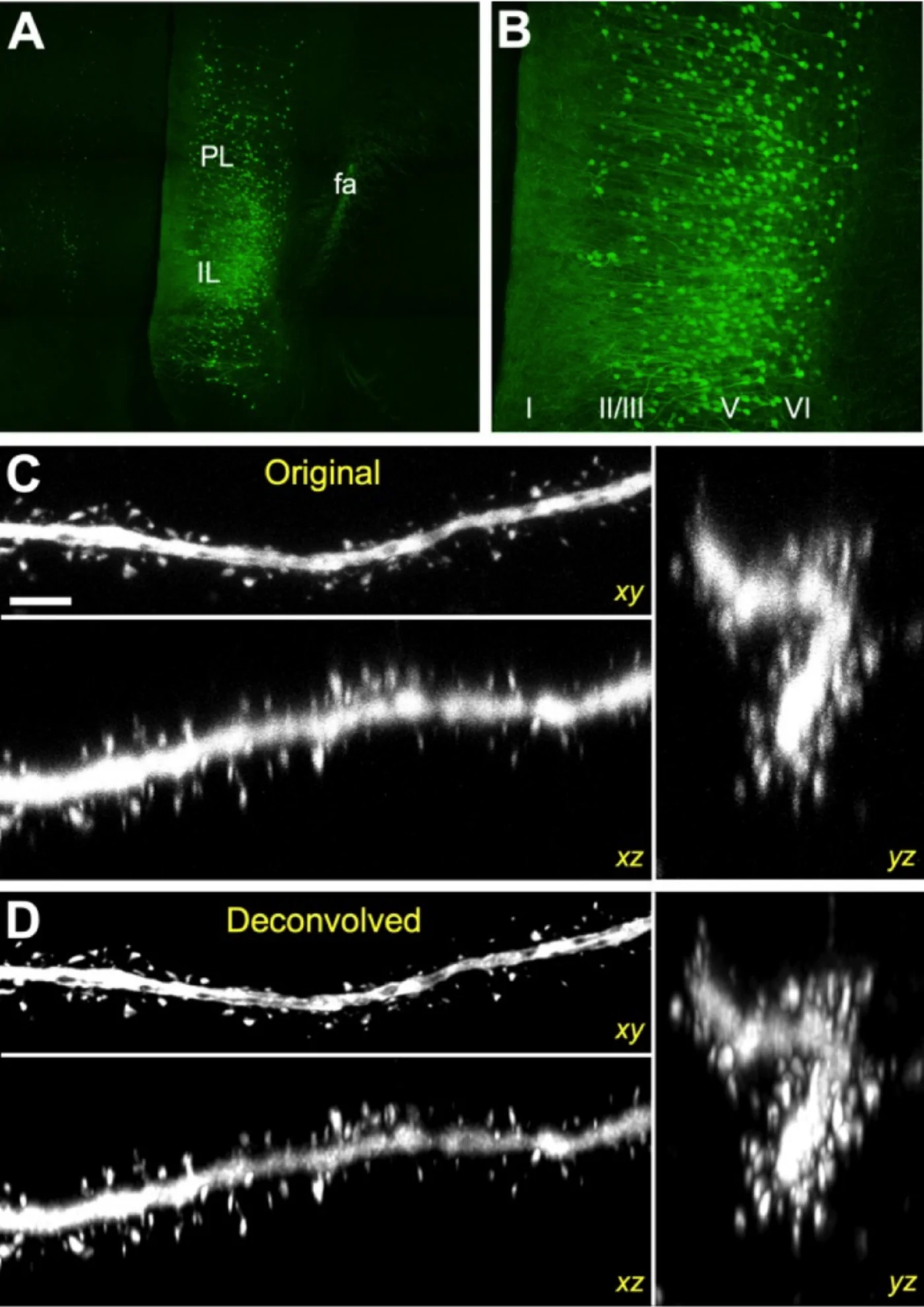
mGreenLantern labeling and high-resolution imaging of dendritic spines in vmPFC to BLA/BMA projection neurons. **a** Low-magnification epifluorescence image showing mGreenLantern-expressing neurons in infralimbic cortex (IL) and adjacent prelimbic cortex (PL). fa, forceps anterior, corpus callosum. **b** Higher magnification view showing labeled pyramidal neurons across cortical layers II/III, V, and VI. **c** High-resolution confocal image of a representative dendritic segment before deconvolution processing, shown in xy (top left), xz (bottom left), and yz (right) planes. **d** Same dendritic segment after Huygens deconvolution processing, illustrating improved resolution and contrast for three-dimensional spine reconstruction and morphometric analysis. Scale bar in **c** represents 500 μm (**a**), 100 μm (**b**), and 5 μm (**c**,** d**)

To enable visualization of dendritic spine morphology in vmPFC→BLA/BMA neurons, we used an intersectional viral strategy combining retrograde Cre-expressing virus targeting the basal amygdala, in conjunction with viral injection of a Cre-dependent mGreenLantern in vmPFC (Fig. 6). This approach produced robust labeling of vmPFC pyramidal neurons projecting to the BLA/BMA, with clear visualization of dendritic arbors and individual dendritic spines (Fig. 7a, b). High-resolution confocal imaging followed by deconvolution processing enabled detailed three-dimensional reconstruction and morphometric analysis of dendritic spines along both apical and basal dendrites of labeled projection neurons (Fig. 7c, d).

**Fig. 8:**
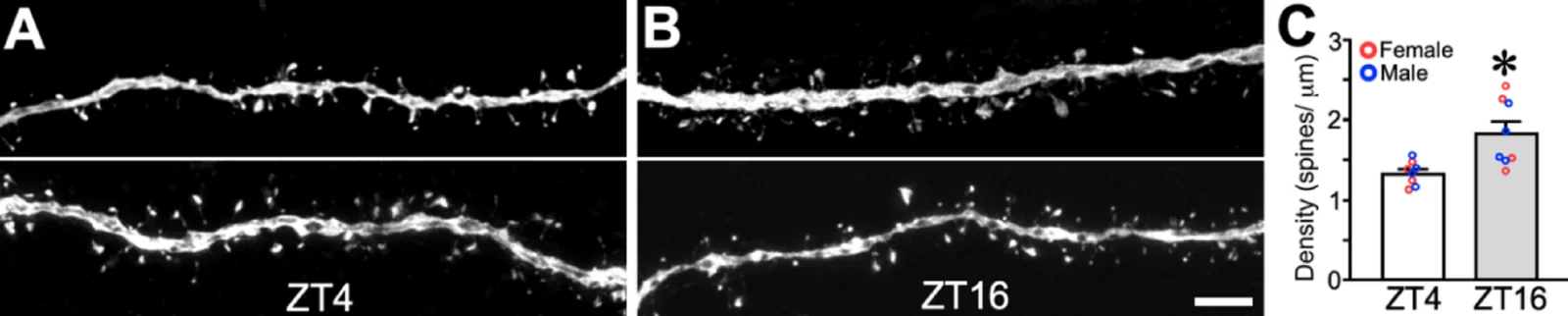
Greater overall dendritic spine density in vmPFC to BLA/BMA projection neurons at ZT16 compared to ZT4. **a**,** b** Representative high-resolution confocal images of dendritic segments from rats perfused at ZT4 (**a**) and ZT16 (**b**). Scale bar = 5 μm. **c** Spine density (*n* = 8 per time-point), showing 34% greater density at ZT16 compared to ZT4. Individual data points are color-coded by sex. *p <0.05

Analysis of overall spine density revealed a significant time-of-day difference (unpaired t-test with Welch’s correction, t_8.7_ = 3.21, *p* =.011, d = 1.60), with 34% greater density at ZT16 (1.84 spines/µm) compared to ZT4 (1.35 spines/µm; Fig. 8a-c).

**Fig. 9:**
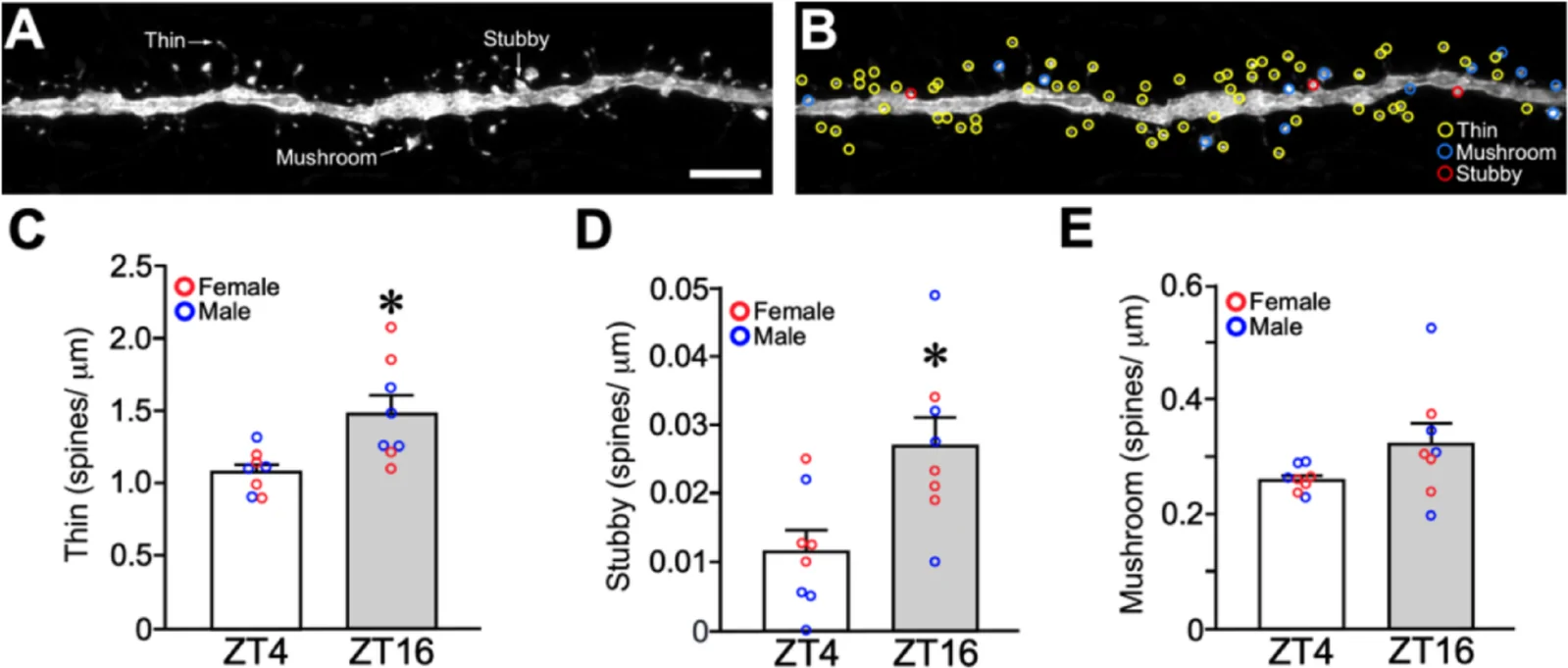
Time-of-day differences in spine density are driven by increases in thin and stubby spine subtypes. **a** Representative dendritic segment illustrating morphometric classification of spine subtypes (thin, mushroom, and stubby) based on head-to-neck diameter ratios and spine length measurements. **b** Same segment with color-coded spine classifications (yellow = thin, blue = mushroom, red= stubby). Scale bar = 5 μm. **c**–**e** Spine subtype densities for thin (**c**), stubby (**d**), and mushroom (**e**) spines at ZT4 and ZT16 (*n* = 8 per time point). Both thin and stubby (p =.009 for each) spine densities were significantly greater at ZT16, whereas mushroom spine density showed no significant time-of-day difference. *p<0.05

Dendritic spines exhibit distinct morphological subtypes that have been associated with different functional properties. To determine which spine subtypes contributed to the overall increase in density at ZT16, we classified individual spines as thin, mushroom, or stubby based on morphometric criteria including head diameter-to-neck diameter ratios and spine length-to-neck diameter ratios (Fig. 9a, b). Analysis of spine subtype densities revealed that both thin (t_14_ = 3.06, *p* =.009; d = 1.53; Fig. 9c) and stubby (t_14_ = 3.01, *p* =.009; d = 1.51, Fig. 9d) spine densities were significantly greater at ZT16 compared to ZT4. By contrast, mushroom spine density showed no significant time-of-day difference (t_14_ = 1.76, *p* =.10; Fig. 9e). Across both time-points, thin spines were the most abundant subtype, followed by mushroom spines and stubby spines, consistent with previous reports of spine subtype distributions in prefrontal cortical pyramidal neurons (Anderson et al. 2016, 2020; Baratta et al. 2019).

#### Sex-specific patterns in dendritic spine volume distributions

**Fig. 10:**
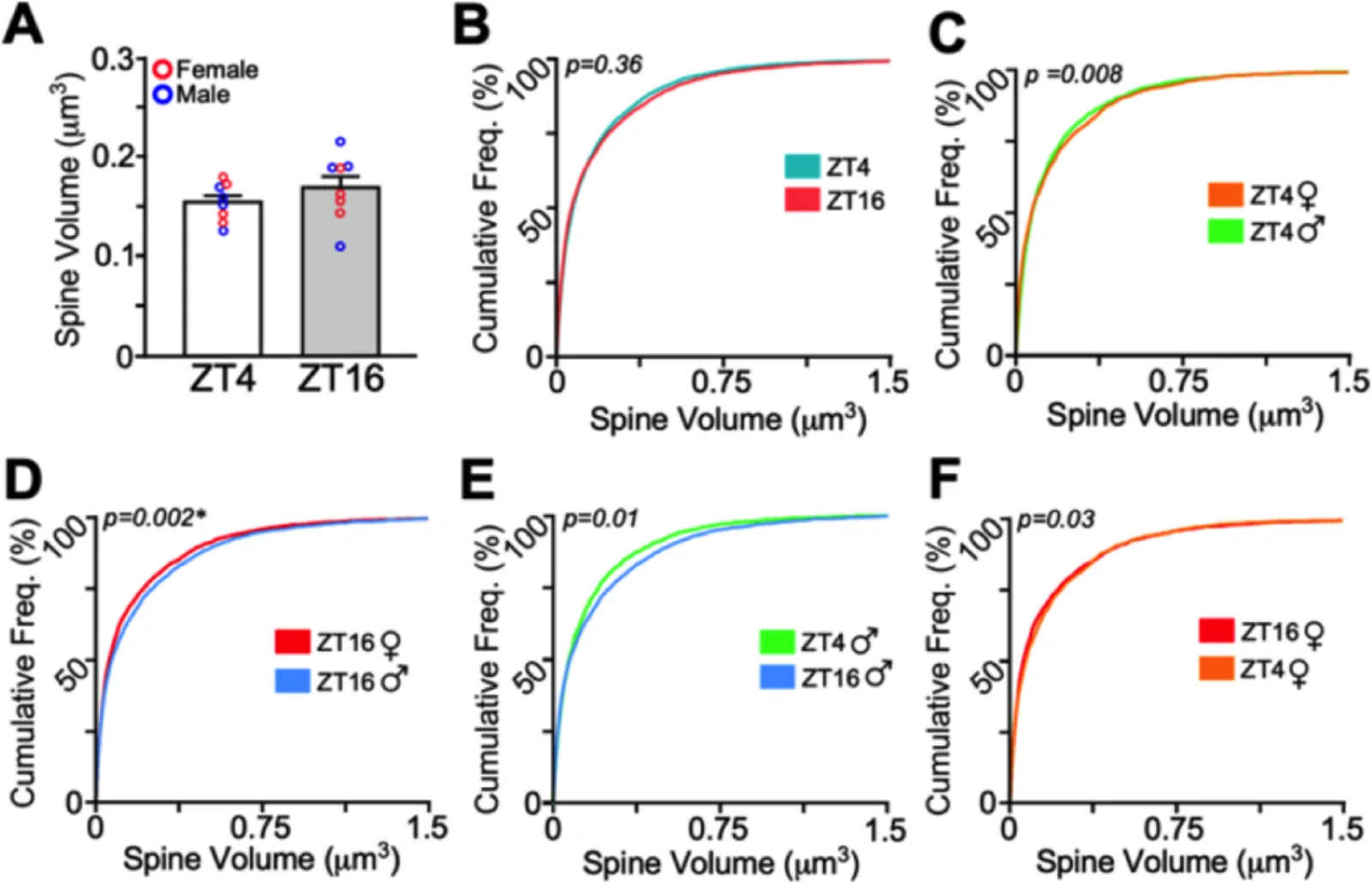
Sex-specific patterns in dendritic spine volume distributions. **a** Mean spine volumes at ZT4 and ZT16 collapsed across sex, showing no significant time-of-day effect. **b** Cumulative frequency distributions of spine volumes for ZT4 (cyan) and ZT16 (pink) with sexes combined, showing no significant difference. **c** Cumulative frequency distributions separated by sex at ZT4, showing females (orange) exhibited a trend toward greater representation of large-volume spines compared to males (green; *p* =.008). **d** Cumulative frequency distributions separated by sex at ZT16, showing males (blue) exhibited significantly greater representation of large-volume spines compared to females (red; D = 0.055, *p* =.002). **e**,** f** Cumulative frequency distributions for females and males, showing a nominal rightward shift in males (**e**), and minimal distributional changes in females (**f**), across time-points. *p <.0025, Bonferroni-corrected threshold for K-S tests (α = 0.01 with 4 comparisons)

Mean spine volumes were similar between ZT4 (0.15 ± 0.01 μm³) and ZT16 (0.17 ± 0.01 μm³; t_14_ = 1.01, *p* =.33) Fig. 10a and population analysis of cumulative frequency distributions revealed no significant time-of-day effect when sexes were combined (K-S test: D = 0.025, *p* =.15) Fig. 10b. Interestingly, however, analysis by sex revealed opposing patterns that account for the lack of overall time-of-day effect. At ZT4, females exhibited a trend toward greater representation of large-volume spines compared to males (D = 0.055, *p* =.008) Fig. 10c. This pattern reversed at ZT16, with males showing significantly greater representation of large-volume spines than females (D = 0.055, *p* =.002) Fig. 10d. Only the ZT16 sex comparison survived correction for multiple comparisons (Bonferroni-corrected threshold: *p* <.0025 for α = 0.01 with 4 comparisons).

Analysis of time-of-day effects within each sex revealed that this reversal was driven primarily by changes in males. Male rats showed a rightward shift in spine volume distribution from ZT4 to ZT16 (D = 0.051, *p* =.012) Fig. 10e, with an increased proportion of large-volume spines at the active phase (> 1.0 μm³: 2.3% at ZT16 vs. 1.1% at ZT4). Female rats showed minimal distributional changes across time points (D = 0.044, *p* =.029) Fig. 10f, with similar proportions of large-volume spines at ZT4 and ZT16 (> 1.0 μm³: 1.6% at both time-points). However, neither within-sex time comparison survived correction for multiple comparisons. Collectively, these findings indicate that while the primary circadian effect on vmPFC to BLA/BMA projection neurons is increased spine density in both sexes, males additionally exhibit an enrichment of large-volume spine phenotypes at the active phase.

## Discussion

The circadian system dynamically regulates all biological process, thereby ensuring optimal function throughout the fluctuating demands of each day (Bass and Lazar 2016; Koronowski and Sassone-Corsi 2021). Time-of-day is an important biological variable for biomedical research, as it can reveal new mechanistic insights underlying optimal function of various biological processes (Nelson et al. 2022). In this study we extend our previous findings of time-of-day regulation of auditory conditioned fear extinction memory and we reveal functional and structural correlates in a vmPFC to amygdala neuronal pathway known to be important for conditioned fear extinction learning (Milad and Quirk 2012).

In our conditioned fear experiment we found that female rats trained and tested during their circadian active phase (ZT16) had reduced freezing to the tone on Day 3 extinction testing, suggesting that these rats had superior conditioned fear extinction memory compared to rats trained and tested during their circadian inactive phase (ZT4). This result replicates our previously published findings in male rats (Woodruff et al. 2015, 2018; Hartsock et al. 2023, 2024) and demonstrates that this process is also robust in female rats. We note, however, that during Day 1 fear conditioning the ZT16 rats also exhibited less freezing than ZT4 rats during the first tone-shock intertrial interval and the second tone presentation. ZT16 rats also exhibited less freezing than ZT4 rats during the pre-tone baseline period of Day 2 testing, suggesting that ZT16 rats had less generalization of conditioned fear to Context B and/or the general test situation. Thus, we cannot rule out the possibility that the pronounced time-of-day difference in extinction recall on Day 3 reflects, at least in part, a resistance of rats trained and tested at ZT16 to the full extent of the initial conditioned fear learning. Nevertheless, on Day 2, ZT16 rats exhibited a strong conditioned fear response to the initial tone presentations in context B that was comparable to the response of ZT4 rats, and both ZT16 and ZT4 rats had similar within session rates and magnitude of fear extinction on Day 2. Furthermore, both groups of rats exhibited very little fear responses during the pre-tone baseline period on Day 3 testing. We also observed no time-of-day differences in general behavioral activity levels of control rats (no footshock on Day 1) during the identical 3-day testing contexts, ruling out the possibility that time-of-day differences in freezing responses may be related to time-of-day differences in general behavioral activity levels in these test situations.

Another key finding of this study is that rats trained and tested at ZT16 had greater FOS expression in the vmPFC immediately after Day 3 extinction recall testing compared to rats trained and tested at ZT4. This time-of-day difference in FOS expression was also present in IL→BLA/BMA neurons. Although there was not a statistically significant time-of-day by training condition interaction, only fear conditioned rats had time-of-day differences in vmPFC FOS expression as assessed by exploratory follow-up Bonferroni correction multiple-comparisons analysis. Thus, there was support for enhanced increased FOS expression in the vmPFC of rats undergoing conditioned fear extinction recall at ZT16 compared to ZT4. The greater vmPFC FOS expression of ZT16 rats on Day 3 may reflect their superior conditioned fear extinction memory. This increased FOS expression may also reflect enhanced neuroplasticity processes in the vmPFC associated with conditioned fear extinction learning and recall when trained and tested at ZT16 compared to ZT4.

Converging evidence indicates that neural activity within the IL portion of the vmPFC is vital for auditory conditioned fear extinction learning and recall (Milad and Quirk 2012; Tovote et al. 2015; Izquierdo et al. 2016; Bouton et al. 2020). Moreover, a critical aspect of IL activity for extinction learning and recall is increased activity of IL neurons that project to the BLA/BMA (Milad and Quirk 2012; Do-Monte et al. 2015; Bukalo et al. 2015; Adhikari et al. 2015; Kim et al. 2016; Bloodgood et al. 2018). In contrast, PL activity promotes conditioned fear expression (Corcoran and Quirk 2007; Sotres-Bayon et al. 2012; Tovote et al. 2015; Marek et al. 2019), and manipulation of PL activity can produce opposite effects to IL manipulation on conditioned fear extinction memory (Vidal-Gonzalez et al. 2006; Senn et al. 2014). Given the importance of the IL to BLA/BMA pathway for extinction learning, it is noteworthy that in our study there was increased FOS expression within this neuronal pathway during extinction recall at ZT16 compared to ZT4. However, the time-of-day difference in vmPFC FOS expression was not restricted to just this population of neurons. Instead, we observed increased FOS expression in both the IL and PL. Marek et al. (Marek et al. 2018) demonstrated the functional importance of PL projections to the IL for extinction learning, that included increased FOS expression in these PL projection neurons following extinction learning. Thus, the PL and IL may function more as an integrated module for both conditioned fear and extinction learning than two independent entities with opposing actions.

The time-of-day difference in extinction recall may reflect circadian regulation that depends on local circadian clocks within the vmPFC. Woodruff et al. (Woodruff et al. 2018) found that the time-of-day difference in extinction recall is absent in rats that have selective knock-down of core clock gene expression (Per1 and Per2 clock genes) within the vmPFC. The time-of-day difference in extinction recall is also absent in rats that lack a circadian pattern of daily corticosterone hormone secretion that has been shown to be necessary for normal daily vmPFC clock gene expression patterns (Woodruff et al. 2015, 2016, 2018). A local circadian clock within the vmPFC may contribute not only to time-of-day differences in vmPFC neural activity but also neuroplasticity. Neuroplasticity processes within IL neurons are necessary for extinction learning and memory (Santini et al. 2004; Burgos-Robles et al. 2007; Marek et al. 2019).

An underlying neurobiological process that may contribute to time-of-day differences in vmPFC neuroplasticity and associated conditioned fear extinction memory is circadian regulation of vmPFC spine formation and maintenance (Yuste and Bonhoeffer 2001; Holtmaat and Svoboda 2009). In support of this prospect, we found that under basal conditions of untrained rats there was greater spine density in vmPFC→BLA/BMA neurons at ZT16 compared to ZT4. We chose to examine dendritic spine measures in untrained rats because any time-of-day differences observed in behaviorally trained rats would be difficult to disentangle from potential learning-related effects on spine plasticity.

In addition to the increase in spine density during the active phase, we noted robust effects in spine subtypes bearing morphological features often classified as thin or stubby spines (Nimchinsky et al. 2002). The preferential increase in thin and stubby spines at ZT16 is particularly noteworthy, as these spine types are associated with activity-dependent synaptic plasticity and are the primary substrates for learning-induced structural changes (Qiao et al. 2022). Thus, it appears that there is a daily circadian fluctuation in the formation and maintenance of dendritic spines within vmPFC→BLA/BMA neurons. The greater spine density at ZT16 may support greater extinction memory consolidation and subsequent retrieval at this time of day. Conditioned fear extinction learning is associated with an increased rate of spine formation in the mouse association frontal cortex (Lai et al. 2012), and greater spine density in IL is associated with greater conditioned fear extinction retrieval (Moench et al. 2016).

Daily circadian fluctuation in vmPFC→BLA/BMA pyramidal neuron spine density may reflect a more widespread property of neocortical and allocortical pyramidal neurons. Other studies have found a similar time-of-day difference in vmPFC spine density in male rats (Perez-Cruz et al. 2009) and mice (Miller et al. 2022). Ikeda et al. (Ikeda et al. 2015) observed greater spine density at ZT13 in hippocampal pyramidal neurons compared to ZT1-10. Studies of in vivo cortical spine formation and morphological characteristics also find time of day differences that include greater spine formation during the active phase in immature mouse cortex (Maret et al. 2011), greater activity-dependent motor cortex spine formation when motor task training occurred during the early portion of the active phase (Liston et al. 2013), and circadian regulation of cortical dendritic spine morphological changes (Jasinska et al. 2015, 2019). Notably, the current study extends these findings by demonstrating circadian regulation of spine density in a functionally defined projection population (vmPFC→BLA/BMA neurons), which may be more directly relevant to understanding circuit-specific mechanisms underlying time-of-day differences in extinction learning.

Our experimental design was powered to detect the time-of-day effect rather than sex differences (*n* = 4/sex/time-point), and consequently, we lack the power to draw firm conclusions as to whether there were time-of-day sex differences in spine density. On the other hand, the unit of analysis for the spine volume measures was the individual spine, which permitted exploratory consideration of sex as a variable. The sexes differed in opposite directions at the two time-points: females trended toward more large-volume spines at ZT4, whereas males showed significantly more at ZT16. This appeared driven largely by males, which shifted rightward in their spine volume distribution from ZT4 to ZT16, whereas females changed minimally. These exploratory observations raise the possibility that males and females rely on partially distinct structural correlates—density increases in both sexes, with an additional shift toward larger spines in males. Testing this directly will require a study powered for sex comparisons that also accounts for ovarian cycle stage.

### Concluding statement

This study provides additional support for the importance of circadian factors to regulate auditory conditioned fear extinction memory. From a translational perspective, the conditioned fear paradigm has provided valuable insights into neurocircuit adaptive mechanisms underlying certain psychological disorders such as depression, anxiety and posttraumatic stress disorder that are associated with impaired conditioned fear extinction (Milad and Quirk 2012; Wurst et al. 2021). It is noteworthy that circadian dysregulation is also strongly associated with these disorders (Agorastos et al. 2020; Leeuw et al. 2023). Further study of the mechanistic basis of circadian regulation of conditioned fear extinction learning may provide new therapeutic insights for addressing these disorders. These studies may also reveal new mechanisms by which the circadian system more broadly regulates learning and memory processes (Hartsock and Spencer 2020).

## Data Availability

The datasets generated during the current study are available upon request to the corresponding author: Robert.spencer@colorado.edu.
